# Exogenous Melatonin Promotes the Salt Tolerance by Removing Active Oxygen and Maintaining Ion Balance in Wheat (*Triticum aestivum* L.)

**DOI:** 10.3389/fpls.2021.787062

**Published:** 2022-01-31

**Authors:** Zhihui Zhang, Liantao Liu, Hongye Li, Shaocong Zhang, Xiaoyi Fu, Xiuzhen Zhai, Na Yang, Jiaming Shen, Ruiqi Li, Dongxiao Li

**Affiliations:** ^1^State Key Laboratory of North China Crop Improvement and Regulation, Key Laboratory of Crop Growth Regulation of Hebei Province, College of Agronomy, Hebei Agricultural University, Baoding, China; ^2^National Engineering Laboratory for Crop Molecular Breeding, National Center of Space Mutagenesis for Crop Improvement, Institute of Crop Sciences, Chinese Academy of Agricultural Sciences, Beijing, China; ^3^Shijiazhuang Academy of Agricultural and Forestry Sciences, Shijiazhuang, China

**Keywords:** salt stress, melatonin, wheat, germination, antioxidative activity

## Abstract

Melatonin (MT) is a small molecule indole hormone that plays an important role in the regulation of biological processes and abiotic stress resistance. Previous studies have confirmed that MT promotes the normal development of plants under stress by mediating physiological regulation mechanisms. However, the physiological mechanism of exogenous MT regulating seed germination and seedling growth of wheat under salt stress is still unclear. In this study, NaCl stress decreased germination rate and inhibited seedling growth of wheat, but shoot length, root length, and plant weight of SM15 did not change significantly. The addition of 300 μM MT in the cultivation solution directly promoted the germination rate of SM15 and ZM18, and lateral root production, but decreased the germination rate of JM22 and inhibited the length of germ and radicle of three varieties under salt stress. For wheat seedling, application of MT could increase proline content, soluble protein, soluble sugar, Ca^2+^ content, and vital amino acid content in leaves to keep high water content, low level of H_2_O_2_ content, and low [K^+^]/[Na^+^] ratio. MT increased root vigor and [K^+^]/[Na^+^] ratio and decreased H_2_O_2_ content in root induced by salt stress. In conclusion, MT enhanced salt tolerance in wheat seeds and seedlings by regulating the synthesis of soluble protein and sugar, ion compartmentation in roots and leaves, enhancement of enzymatic systems, and changes in amino acid levels. Salt resistance varied with different varieties under the same environmental condition. SM15 was a higher salt-resistant variety and JM22 was a salt-sensitive one. In wheat production, the application of exogenous MT should consider the differences among varieties of wheat during the sowing and seedling stages.

## Introduction

Soil salinization, a geologically environmental problem all over the world, is one of the main factors resulting in the decrease in grain production (Liang et al., 2018). According to incomplete statistics, the salinization of arable land globally is increasing at a rate of 0.3–1.5 million hectares year^–1^ (Harper et al., 2021). The total area of salinized soil in China is about 99.13 × 10^6^ hm^2^, which accounts for 1.03% of the total Chinese land area (Yang and Wang, 2015). It is expected that 50% of arable land will be under saline-alkali stress by 2050 due to environmental pollution, lack of freshwater, improper irrigation methods, and other factors (Dou et al., 2019; Yang et al., 2021). Wheat (*Triticum aestivum* L.), one of the medium salt-tolerant crops and the second-largest food crop in the world, also faces soil salinization in its growing area. Salt stress leads to the significant inhibition of seed germination and seedling growth (Shahi et al., 2015). Under salt stress, excessive reactive oxygen species (ROS) are accumulated which will cause cell damage, DNA damage, and oxidative stress to plants, consequently disrupting the physiological balance and leading to reduced photosynthesis and production yield (Zhang et al., 2016; Tripathi et al., 2020). High Na^+^ accumulation in plant cells leads to osmotic stresses and limits uptake and utilization of other nutrition ions, which causes moisture loss and electrolytic leaching due to cell membrane damage (Abbas et al., 2015; Ahmed et al., 2015; Rebey et al., 2017).

Melatonin (MT), an amine hormone, has been confirmed extensively for enhancing plant tolerance to various abiotic stresses (Zhang et al., 2013; Turk et al., 2014; Ren et al., 2020). There are multiple mechanisms verified in MT helping plants to enhance abiotic stress tolerance (Huangfu et al., 2021; Sun et al., 2021). The application of MT has shown stimulating antioxidant enzymes and scavenging ROS in plants under stress conditions (Park et al., 2013; Li et al., 2018), preventing chlorophyll degradation and increasing photosynthetic efficiency, reducing ion toxicity (e.g., heavy metals, Na^+^, and so on) and increasing osmotic substances to maintain water status (Gao et al., 2016; Cai et al., 2017; Siddiqui et al., 2019a,^2020^; Al-Huqail et al., 2020), and enhancing secondary metabolite biosynthesis and upregulating defense genes to decrease cell injury (Li H. et al., 2017; Zhao et al., 2017; Elsayed et al., 2021). Under salt stress, MT alleviates directly ROS burst and cell damage for bermudagrass (Shi et al., 2015). Also, MT alleviates the inhibitory effects of NaCl stress on germination of cucumber seeds mainly by increasing antioxidant enzymes, affecting phytohormone biosynthesis and catabolism, and regulating storage protein degradation to vital amino acids during seed germination (Zhang H. J. et al., 2014; Zhang et al., 2017). MT has been reported to extend longevity by significantly reducing chlorophyll degradation, delaying the leaf senescence, and preventing cell death in rice plant and tomato seedlings under salt stress (Siddiqui et al., 2019b; Zhao et al., 2021). During wheat seedling growth, MT accelerates the transformation from arginine and methionine to polyamines in wheat seedlings (Ke et al., 2018). Salt tolerance enhanced in maize with MT is most likely due to the improvement in photosynthetic capacity, antioxidative capacity, and ion homeostasis (increased K^+^ contents and K^+^/Na^+^ ratios) in leaves (Jiang C. et al., 2016). Also, Liu et al. (2020) demonstrate that MT improves salt tolerance in rice by reducing K^+^ efflux induced by high NaCl concentrations in roots. Subsequent researches have partly revealed the mechanisms of MT, which affects directly or indirectly salt resistance in many plants. However, MT application had no effects on the growth of wheat and maize under normal conditions (Ke et al., 2018; Ren et al., 2020). It has not fully elucidated a universal pathway on whether or how MT launches crosstalk with vital regulators under stress.

Different concentrations of MT may have differential effects in mediating abiotic stress defense. MT at 200 μmol L^–1^ can effectively inhibit ROS production, improve the biomass and chlorophyll content, and regulate the photosynthetic characteristics of cotton seedlings under salt stress (Jiang et al., 2021). MT at 300 μmol L^–1^ alleviates the negative effect of water stress on wheat germination and promotes morphological development including radicle length, radicle number, and plumule length of germinated seeds (Li et al., 2020). About 800 μmol L^–1^ MT used for priming seed significantly improves germination energy, germination percentage, proline content, and total phenolic content of maize (Jiang X. et al., 2016). Application of exogenous MT (100 μmol L^–1^) alleviates ROS burst and protects the photosynthetic activity in maize seedlings under salt stress (Chen et al., 2018). For some other reports, MT at a concentration of 100 μmol L^–1^ inhibits seed germination and seedling growth and enhances the toxic effect of copper on seedlings, but 1 or 10 μmol L^–1^ MT can eliminate the inhibitory effect of copper on the fresh weight of red cabbage seedlings (Posmyk et al., 2008). Pretreatment with 1 μmol L^–1^ MT was also found to partially mitigate the inhibition of shoot dry weight induced by salt stress (Ke et al., 2018). Li X. et al. (2017) showed that 10–500 μmol L^–1^ MT could help to recover seed germination potential, germination index, and vigor index on two varieties of rice treated with 120 mM NaCl to control levels. It seems that MT can regulate and enhance stress resistance by multiple mechanisms in a concentration-dependent manner.

In wheat production, different varieties show different resistances, adaptive areas, and production yields. So far, the roles of MT in mediating salt stresses on physiological regulation still need to be explored in different wheat varieties. In this study, seed germination and seedling growth of winter wheat experiments were conducted in a hydroponic solution with NaCl (100 mM) or without NaCl (100 mM), and the addition of MT. The purposes of this study were to (1) verify that MT enhances seed germination rate and antioxidant activities of seedling, (2) elucidate the regulatory effect of MT on ion exchange in the organelles, and (3) compare the regulatory effects of MT on the different varieties of wheat under salt stress. The results of this experiment provide theoretical support to promote the application of exogenous MT on saline-alkali wheat to increase arable land reserve and improve food security.

## Materials and Methods

### Plant Material

The experiment was conducted in 2021 at the Hebei Agricultural University (38° 85′ N, 115° 30′ E), Hebei Province, China. Three commercial wheat (*T. aestivum* L.) cultivars, ‘Jimai 22’ (JM22), ‘Shimai 15’ (SM15), and ‘Zhoumai 18’ (ZM18), were used in this study. All varieties are high-yielding and extensively cultivated in Huang-Huai-Hai region. ZM18 is suitable for planting in south section of the Huanghuai winter wheat area, and JM22 is suitable for planting in north section of the Huanghuai winter wheat area. SM15 has comprehensive resistance and good adaptability.

### Experimental Design

This experiment was performed in a growth chamber.

#### Seed Germination

In total, 450 seeds of each wheat variety were surface-sterilized with 75% ethanol for five minutes. After that, all surface-sterilized seeds were washed thoroughly with distilled water and were transferred into a glass plate for germination. Totally, two layers of filter paper saturated with three different treatment solutions: CK (distilled water), NaCl (100 mM), and NaCl-MT (100 mM NaCl + 300 μM MT) were placed in each plate. The concentrations of NaCl and MT solutions were selected referring to Li X. et al. (2017), Ke et al. (2018), and our former study (Li et al., 2020). The germination conditions were set at a light/dark shift (12/12h). The day/night temperature was 20/15°C. Light intensity was 600 μmol/m^2^/s^1^. Relative humility was 60%. The number of seed germinated was recorded daily till the 10th day. Water and other solutions were added into the germination plates to keep suitable humidity.

### Hydroponic Experiments

At first, some other healthy seeds of three wheat varieties were germinated in a hole tray with moist vermiculite. The indoor environmental conditions were the same as those described above for the germination plate experiment. When the second leaf appeared, seedlings were removed out of hole tray gently and washed off vermiculite attached to the roots in tap water several times. Then, seedlings were transferred into Hoagland nutrient solution in plastic boxes covered with black film to avoid light. After about 1 week of recovery growth, seedlings were treated with fresh Hoagland nutrient solution as control, 100 mM NaCl solution as salt stress, and 100 mM NaCl + 300 μM MT. Each treatment was repeated three times. After 24 h, leaf and root samples were collected and quickly frozen using liquid nitrogen and then stored in a −80°C freezer until the measurements were taken.

### Measurement of Morphological Parameters

Germinated seeds were recorded daily when radicle length exceeded 2 mm according to Liu et al. (2016). Germ and radicle length and radicle number of 10 randomly selected grains from each treatment were recorded with a ruler for 24 h after treatment.

Shoot and root length of five randomly selected seedlings from each replication were measured 24 h after treatment. Seedlings of each treatment with triplicates were dissected into roots, stems, and leaf and their fresh weights were recorded immediately. Then, the weighed fresh samples were kept in the paper bags to be dried in an oven at 105°C for 30 min and then at 75°C until the consistent weight was obtained.

### Na^+^, K^+^, and Ca^2+^ Contents

Na^+^, K^+^, and Ca^2+^ contents of the whole-plant tissues were determined using a previous method with minor modifications (Chen et al., 2017). After the measurement of dry weight, samples were digested in a mixed solution of perchloric acid or concentrated nitric acid (volume ratio, HClO_4_:HNO_3_ = 1:5, v:v) in a glass test tube. More solution has replenished the solution to 12 mL. The ion content of a solution in a glass test tube was extracted in boiling water bath for 5 h and measured using an atomic absorption spectrophotometer (ZA-3000; Hitachi Instruments, Tokyo, Japan).

### Hydrogen Peroxide Content and Detection of H_2_O_2_ and O_2_^–^

Hydrogen peroxide (H_2_O_2_) content in the leaf and root samples was determined using a H_2_O_2_ assay kit (A064, Nanjing Jiancheng Bioengineering Institute, Nanjing, China) according to the manufacturer’s user manual. H_2_O_2_ accumulation in root samples was visualized according to what Liu (2020) described. About 1.5 cm root tip was soaked and stained in 1% (w/v) 3,3′-diaminobenzidine staining (DAB, dissolved with 10 mM MES buffer on pH value = 6.5) and incubated in dark at 25°C for 8 h. Then, the samples were washed several times and observed using an optical microscope (Leica, Wetzlar, Germany).

Superoxide anion (O_2_^–^) accumulation in root samples (about 1.5 cm) was also visualized according to Liu’s (2020) method. Root samples were stained in 100 μM nitro blue tetrazolium chloride (NBT) dissolved with 50 mM phosphate buffer (pH value = 6.4) for 15 min and observed using an optical microscope (Leica, Wetzlar, Germany).

### Antioxidant Enzymes, Soluble Sugar, Soluble Protein, and Malondialdehyde Content

Fresh leaf sample (0.5 g each) was triturated in 50 mM phosphate buffer (pH = 6.4). Homogenate solution was centrifuged at 20,000 *g* at 4°C for 15 min. Superoxide dismutase (SOD), peroxidase (POD), catalase (CAT) activity, soluble sugar, soluble protein, and malondialdehyde (MDA) contents were determined using assay kits (A064, Nanjing Jiancheng Bioengineering Institute, Nanjing, China) according to the manufacturer’s instructions. Soluble sugar and protein contents were determined using a soluble sugar assay kit (A145-1-1, Nanjing Jiancheng Bioengineering Institute) and a BCA assay kit (BCAP-1-W, Suzhou Comin Biotechnology Co., Ltd.) according to the manufacturer’s user manual.

### Extraction and Measurement of Amino Acid Content

#### Sample Preparation

The leaf and root samples (0.20 g) were accurately weighed and placed in the sample bottle (12 mL), into which 10 mL 6 mol/L hydrochloric acid (containing 0.1% phenol) was added. The mixed solution was homogenized by ultrasound. The samples were hydrolyzed in the oven at 110°C for 24 h. After cooling, the filtrate was mixed and filtered by a 0.45 μm water membrane. One milliliter of the filtrate was put into a rotary evaporator and dried at 80°C. Finally, 2.00 mL 0.1 mol/L hydrochloric acid was added to evaporated samples in the rotary evaporator and then mixed evenly by the whirlpool and transferred to the sample bottle for further use.

#### Standard Curve Development

Seventeen amino acid standards including aspartic acid (Asp), glutamic (Glu), histidine (His), serine (Ser), arginine (Arg), glycine (Gly), threonine (Thr), proline (Pro), alanine (Ala), valine (Val), methionine (Met), cysteine (Cys), isoleucine (Ile), leucine (leu), phenylalanine (Phe), lysine (Lys), and tyrosine (Tyr) were calculated and accurately weighed in a volumetric flask. The mixed standard was diluted using 0.1 mol/L hydrochloric acid to obtain a final concentration of 500 mg/L of each amino acid. An appropriate amount of 500 mg/L mixed standard was diluted to make 10, 25, 50, 100, 150, 200, 300, and 400 mg/L mixed standard solution. The gradient elution procedures are shown in Table 1.

**TABLE 1:** Gradient elution program.

Time (min)	Mobile phase (%)	
	A	B
0	18	82
10	18	82
15	29	71
25	34	66
30	55	45
37	60	40
39	18	82
45	18	82
60	18	82

#### Aminoacyl Derivatization

Three solutions including 100 μL mixed standards, 200 μL buffer solution (0.5 mol/L sodium bicarbonate solution, 0.5 mol/L sodium carbonate solution, pH value = 9.0), and 100 μL 2,4-dinitrochlorobenzene (100 mg/mL) were swirly mixed and then reacted at 90°C for 90 min in dark. Thereafter, 50 μL of 10% acetic acid solution and 550 μL ultrapure water were added to 1.00 mL. The mixed solution was swirled and mixed and then filtered with 0.45-μm organic film and filter liquor prepared for measuring.

#### Chromatographic Analysis

An high-performance liquid chromatography (HPLC, Agilent 1200, United States) equipped with a C18 column (4.6 mm × 250 mm, 5 μm; Kromat Universil, catalog no. 35D5) and a diode array detector was used. The column temperature was 40°C. Mobile phase A was pure acetonitrile, and mobile phase B was acetic acid – sodium acetate buffer solution (2.50 g/L sodium acetate, 1.17 mL/L glacial acetic acid, 1.50 mL/L triethylamine, pH value = 5.25 ± 0.05). The flow rate was 1 mL/min. The sample injection amount was 10 μL with 10- to15-min intervals. The detection wavelength was 360 nm.

### Statistical Analyses

All data were processed using analysis of variance (ANOVA) in triplicate in Excel 2003 and IBM SPSS Statistics 17.0 (IBM Corp., Armonk, NY, United States). Duncan’s new multiple range (DMR) test at a 5% probability level was used to test the differences among the means. Significant differences were labeled based on DMR.

## Results

### Exogenous Melatonin Promotes Wheat Germination Trait

**FIGURE 1:**
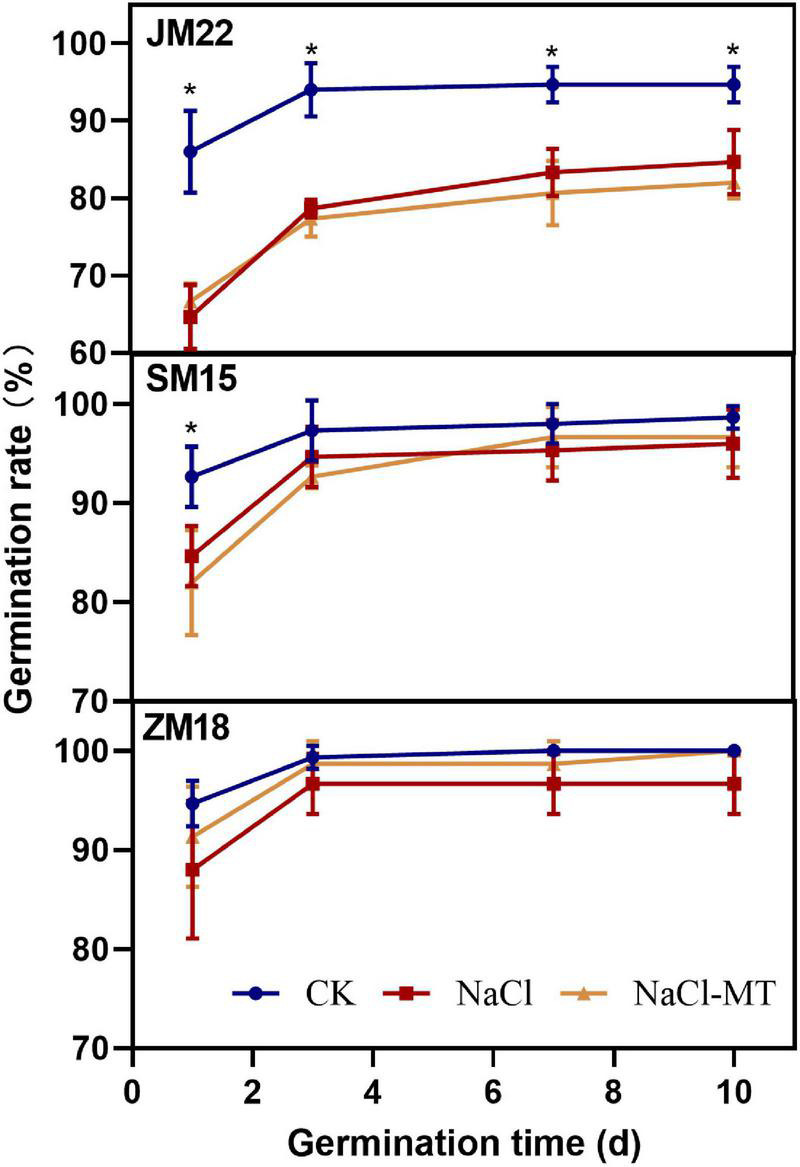
Effects of MT on germination trait under salt stress. For each trait, asterisks represent significantly different according to Duncan’s test at a *p* < 0.05 threshold.

The effect of MT on wheat germination rate under salt stress is shown in Figure 1. The germination rate of CK reached the highest value on the third day and then showed a stable performance. The germination rate of wheat treated with NaCl increased rapidly in the first 3 days and then showed differences: JM22 increased steadily, and SM15 and ZM 18 were basically stable. The germination rate of wheat decreased significantly under salt stress, especially for Jimai22, which was 11.09% lower than CK (*p* < 0.05), but there was no significant difference between Shimai 15 and Zhoumai18.

### Exogenous Melatonin Inhibits Root Length and Germ Length and Promotes Radicle Number

Radicle number, root length, and germ length were also affected by salt stress significantly (Table 2). NaCl stress significantly decreased the length of germ and root of JM22, SM15, and ZM18 by 23.99 and 40.76, 27.97 and 44.79, and 16.79 and 33.43%, respectively. The radicle number did not change significantly under the NaCl treatment than that under CK. MT application further significantly decreased the length of germ and root of JM22, SM15, and ZM18 by 30.98 and 68.69, 45.41 and 75.29, and 37.66 and 70.27%, respectively. Meantime, the radicle number was significantly increased under NaCl-MT than the other two treatments.

**TABLE 2:** Effects of MT on radicle number, root length, and germ length under salt stress.

Varieties	Treatments	Germ length (cm)	Root length (cm)	Radicle number
Jimai 22	CK	15.17a	22.52a	5.47c
	NaCl	11.53b	13.34c	6.00c
	NaCl-MT	10.47b	7.05d	7.93a
Shimai 15	CK	13.29a	21.77a	5.00b
	NaCl	9.5733b	12.02c	5.07b
	NaCl-MT	7.255c	5.38d	6.55a
Zhoumai 18	CK	13.94a	18.13a	5.00d
	NaCl	11.60c	12.07bc	5.27cd
	NaCl-MT	8.69d	5.39d	7.33a

*For each trait, bars with the same letter are not significantly different according to Duncan’s test at a p < 0.05 threshold.*

### Melatonin Application Relieve Inhibited Effect of NaCl on Shoot and Root Length

**FIGURE 2:**
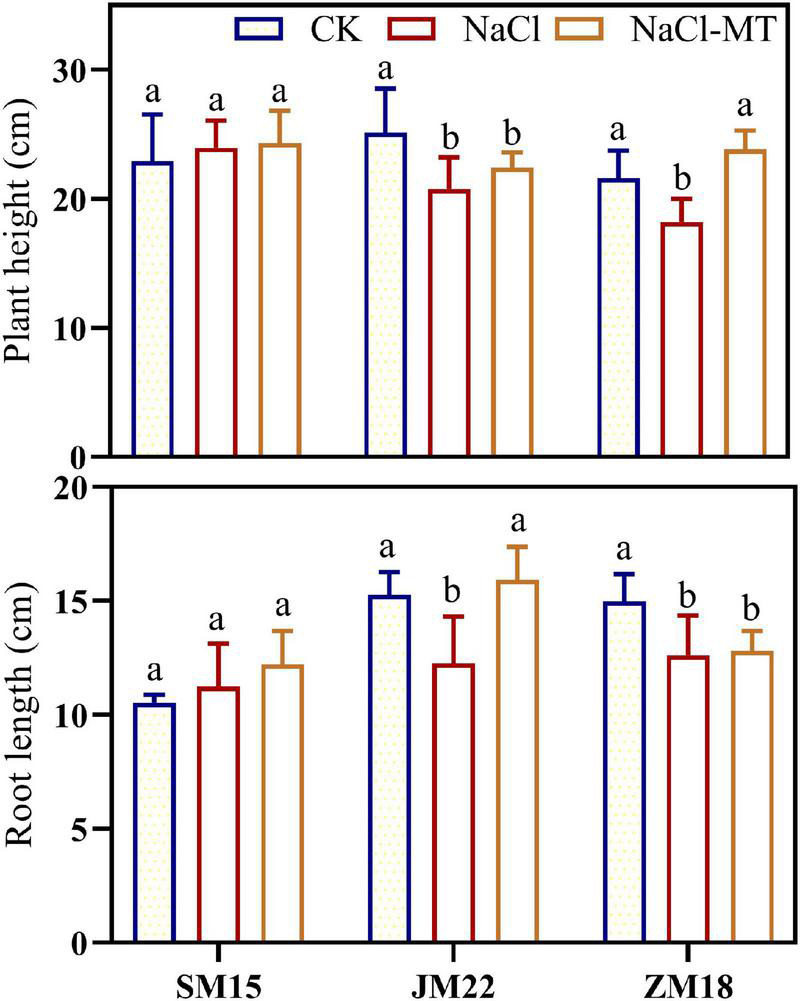
Effects of MT on plant height and root length under salt stress. For each trait, bars with the same letter are not significantly different according to Duncan’s test at a *p* < 0.05 threshold.

Figure 2 shows that shoot and root lengths of JM22 and ZM18 were both decreased significantly under NaCl stress compared with CK. MT application increased those indexes to some degrees, in which the shoot length of ZM18 and root length of JM22 were significantly increased under NaCl treatment than those under the NaCl-MT. Additionally, shoot and root lengths of ZM18 did not change significantly under NaCl and NaCl-MT treatments. These results indicated that SM15 showed higher salt resistance compared with the other two varieties.

### Melatonin Application Increased Water Content in Plant Under NaCl Stress

Table 3 shows that fresh weight, dry weight, and water content in root, stem, and leaf of JM22 and ZM18 significantly decreased under NaCl stress. MT application had increased those indexes to the level of CK. However, for SM15, the fresh weight, dry weight, and water content in plants did not change significantly under the NaCl and NaCl-MT treatments compared with CK. These results also indicated that SM15 contained a good ability on salt resistance.

**TABLE 3:** Effects of MT on fresh weight and dry weight of root, stem, and leaf of wheat under salt stress.

Variety	Treatments	Fresh weight (g/plant)			Dry weight (g/plant)			Water content (g/plant)		
		Root	Stem	Leaf	Root	Stem	Leaf	Root	Stem	Leaf
JM22	CK	1.68c	0.89abcd	1.40ab	0.13bcd	0.15ab	0.26a	1.55c	0.74bc	1.14ab
	NaCl	1.33cde	0.63e	1.00c	0.08e	0.08d	0.15c	1.25cde	0.55d	0.84c
	NaCl + MT	2.07b	1.00abc	1.67a	0.15abc	0.15ab	0.28a	1.92b	0.85ab	1.39a
SM15	CK	1.26de	1.02abc	1.47ab	0.10de	0.14abc	0.24ab	1.16de	0.88ab	1.23ab
	NaCl	1.16e	0.87bcd	1.26bc	0.09de	0.11bcd	0.19bc	1.07e	0.76abc	1.07bc
	NaCl + MT	1.62cd	0.85cd	1.40ab	0.12cde	0.10cd	0.19bc	1.50cd	0.75bc	1.21ab
ZM18	CK	2.50a	1.08ab	1.57ab	0.17a	0.15ab	0.25a	2.33a	0.93a	1.32ab
	NaCl	1.38cde	0.78de	1.01c	0.11de	0.11bcd	0.16c	1.27cde	0.67cd	0.85c
	NaCl + MT	2.35ab	1.09a	1.51ab	0.16ab	0.16a	0.25a	2.19ab	0.93a	1.25ab

*For each trait, bars with the same letter are not significantly different according to Duncan’s test at a p < 0.05 threshold.*

### Melatonin Application Regulates Na^+^, K^+^, Ca^2+^ Compartmentation, and [K^+^]/[Na^+^] at Root and Leaf of Plant

Table 4 shows that K^+^, Ca^2+^, and Na^+^ contents in different organelles and varieties followed different changing patterns. Under NaCl stress, compared with CK, Na^+^ content significantly increased in root, stem, and leaf of three wheat varieties; K^+^ and Ca^2+^ contents were both significantly decreased in root and stem (Ca^2+^ content in the stem of SM15 and ZM18 exception); when K^+^ content increased, Ca^2+^ contents decreased significantly in the leaf of JM 22, but Ca^2+^ increased in the leaf of SM15; both K^+^ and Ca^2+^ in leaf were not obviously changed in ZM18. NaCl-MT treatment significantly decreased Na^+^ contents in root compared with that treated by NaCl alone, but increased in stem and leaf. This suggested that MT took effect first in the root, a higher Na^+^ concentration formed in stem and leaf. Also, MT application further decreased K^+^ content in stem and leaf and increased significantly Ca^2+^ content in the leaf of JM22; it decreased K content in the leaf of SM15 and decreased significantly K^+^ content in stem and increased Ca^2+^ in root and leaf of ZM18. [K^+^]/[Na^+^] ratio in root and stem + leaf was significantly decreased under NaCl stress compared with CK. MT application could increase [K^+^]/[Na^+^] ratio in the root, but decrease this value in stem + leaf.

**TABLE 4:** Effects of MT on K^+^, Na^+^, Ca^2+^, [K^+^]/[Na^+^] in root, stem, and leaf of wheat.

Variety	Treatments	Root			Stem			Leaf			Root	Stem + leaf
					
		K^+^	Na^+^	Ca^2+^	K^+^	Na^+^	Ca^2+^	K^+^	Na^+^	Ca^2+^	K^+^/Na^+^	
JM22	CK	47.77a	3.20c	12.57a	42.38a	3.00c	4.63a	41.95b	2.32c	7.11a	49.71a	16.05a
	NaCl	36.86b	16.23a	5.65b	39.10b	8.35b	3.87c	43.92a	10.32b	6.12b	7.55c	4.46b
	NaCl + MT	33.63b	13.40b	6.14b	35.13c	10.62a	4.43ab	40.85b	14.96a	6.20b	8.36b	2.97c
SM15	CK	46.06a	3.44c	7.62a	40.67a	1.75c	3.32b	41.89a	2.02c	5.77b	44.92a	21.96a
	NaCl	39.10b	16.51a	4.32 b	39.35b	10.62b	4.40 a	42.24a	12.83b	7.54a	7.89c	3.48b
	NaCl + MT	39.18b	10.98b	5.03b	39.21b	11.77a	4.50a	38.34b	16.94a	6.96a	11.90b	2.70c
ZM18	CK	43.04a	2.83	10.59a	42.23a	1.92c	4.40 a	38.78a	1.73c	7.01b	50.94a	22.21a
	NaCl	41.55b	17.09	6.20c	37.35c	6.38 b	4.17 a	38.58a	6.89b	6.81b	8.11c	5.73b
	NaCl + MT	38.10b	11.41	6.94b	39.74b	10.61a	4.51a	38.79a	13.36a	8.63a	11.14b	3.28c

*For each trait, bars with the same letter are not significantly different according to Duncan’s test at a p < 0.05 threshold.*

### Melatonin Application Enhance Activity of Antioxidant Enzymes

**FIGURE 3:**
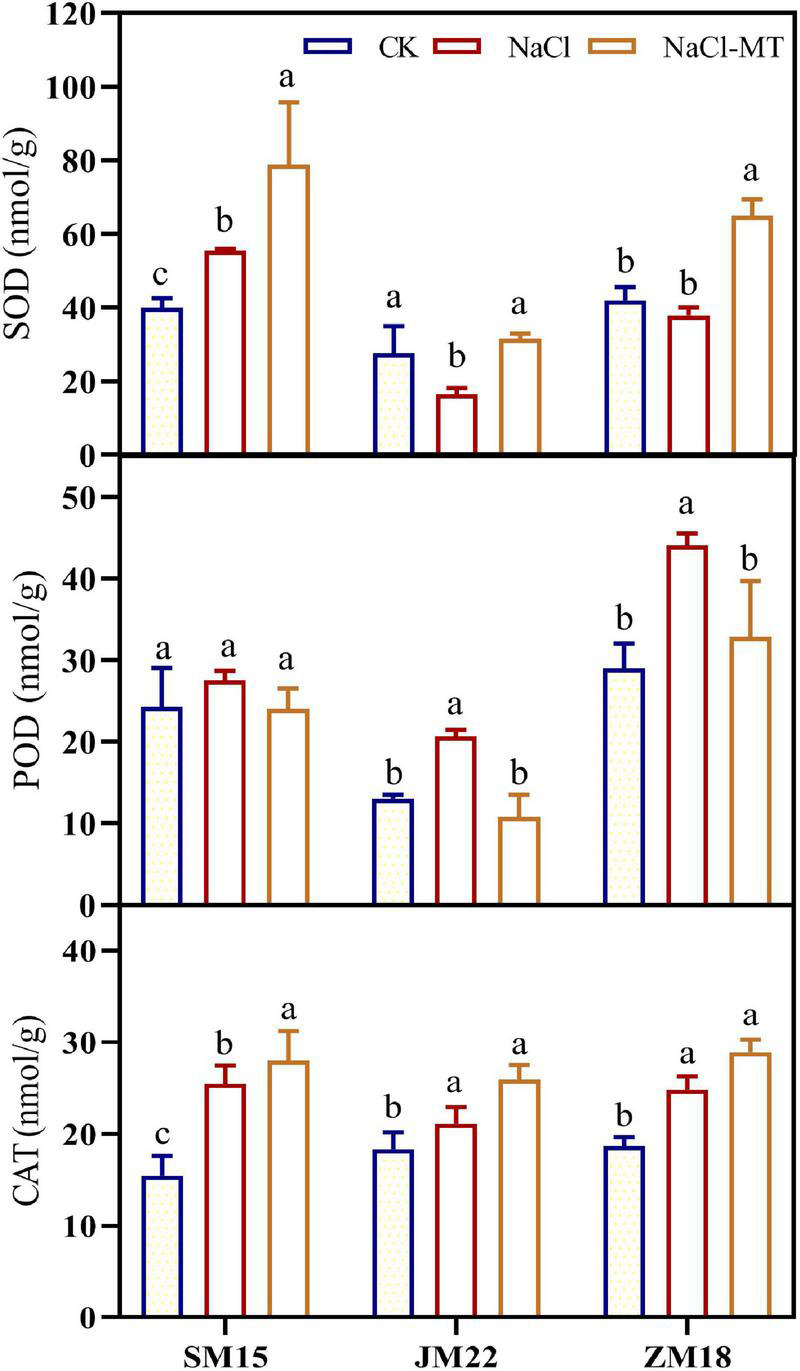
Effects of MT on superoxide dismutase (SOD), peroxidase (POD), and catalase (CAT) under salt stress. For each trait, bars with the same letter are not significantly different according to Duncan’s test at a *p* < 0.05 threshold.

Figure 3 shows that NaCl stress-induced increase of activity for SOD, POD, and CAT in different degrees compared with CK. MT application could enhance further the activity of SOD and CAT in the leaves of three wheat varieties under NaCl treatments; POD activity was recovered to the level of CK. It is noted that SOD activity of JM22 was significantly decreased under NaCl stress, which was possibly explained by the fact that JM22 was salt-sensitive and biochemical characteristics experienced serious damage.

### Melatonin Application Decreased Malondialdehyde Content and Increased Proline Content

**FIGURE 4:**
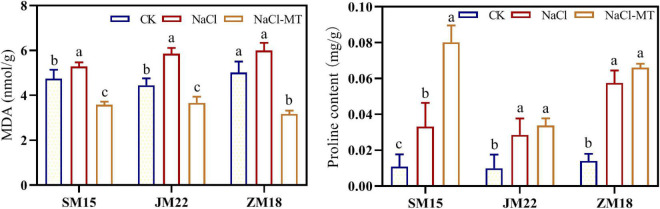
Effects of MT on malondialdehyde (MDA) and proline content under salt stress. For each trait, bars with the same letter are not significantly different according to Duncan’s test at a *p* < 0.05 threshold.

As shown in Figure 4, MDA content and proline content in the leaves of three wheat varieties were both increased under NaCl stress. MT application decreased MDA content under NaCl condition, suggesting that the cell membrane structure had been improved significantly. Additionally, MT further increased the proline content in the leaf of SM15 significantly under NaCl + MT compared with that under the NaCl treatment alone.

### Melatonin Application Increased the Contents of Soluble Protein and Soluble Sugar

**FIGURE 5:**
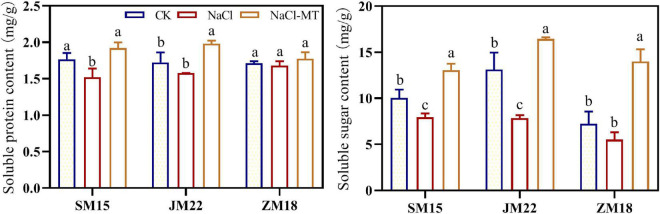
Effects of MT on soluble protein and sugar content under salt stress. For each trait, bars with the same letter are not significantly different according to Duncan’s test at a *p* < 0.05 threshold.

As shown in Figure 5, the contents of soluble protein and soluble sugar were decreased by different degrees under NaCl stress compared with CK. MT application significantly increased the contents of soluble protein and soluble sugar, which helped plants keep good water status under salt stress. Soluble protein content in the leaf of ZM18 did not change significantly under different treatments.

### Melatonin Application Increased the Root Vigor Under NaCl Stress

**FIGURE 6:**
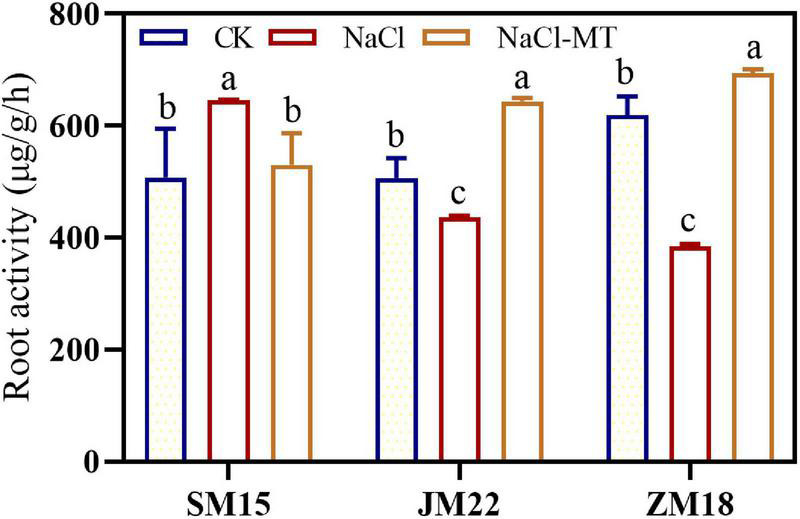
Effects of MT on root activity under salt stress. For each trait, bars with the same letter are not significantly different according to Duncan’s test at a *p* < 0.05 threshold.

Figure 6 shows that the root vigor of JM22 and ZM18 decreased significantly under NaCl stress compared with CK. However, the root vigor of SM15 was increased significantly under NaCl conditions, suggesting higher salt resistance for this variety. MT application had increased the root vigor of JM22 and ZM18 significantly to alleviate the salt damage.

### Melatonin Application Decreased H_2_O_2_ Content in Root and Displayed Less H_2_O_2_ and O_2_^–^ Accumulation in Root

**FIGURE 7:**
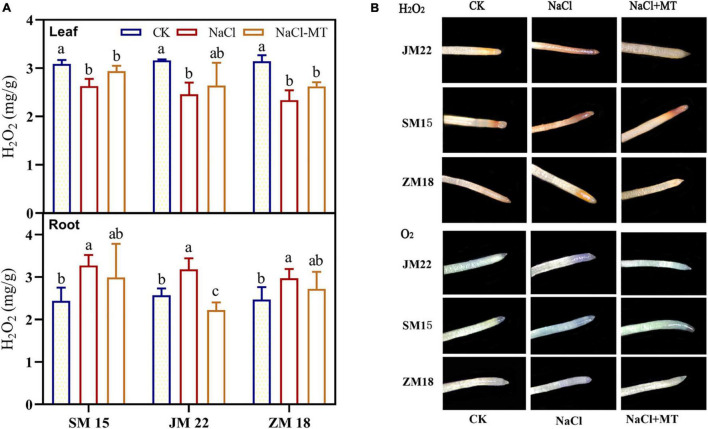
Effects of MT on hydrogen peroxide (H_2_O_2_) content in plant **(A)** and H_2_O_2_ and upper-oxide anions (O_2_^–^) distribution in root **(B)** under salt stress. For each trait, bars with the same letter are not significantly different according to Duncan’s test at a *p* < 0.05 threshold.

Figure 7A shows that H_2_O_2_ contents in root of JM22, SM15, and ZM18 were significantly higher under NaCl stress than that under CK by 23.74, 33.89, and 20.24%, respectively. MT application reduced H_2_O_2_ contents in the roots of three varieties under NaCl stress to the level of CK. H_2_O_2_ and O_2_^–^ accumulation in roots of three varieties with three treatments were visualized by DAB and NBT staining. As expected, the detached roots of MT-treated wheat seedlings displayed less H_2_O_2_ and O_2_^–^ accumulation than those of NaCl-treated seedlings (Figure 7B). However, H_2_O_2_ contents in the leaf of JM22, SM15, and ZM18 were decreased significantly compared with CK. MT application maintained a low level of H_2_O_2_ contents in the leaves of three varieties.

### Melatonin on Cluster Heatmap of Amino Acid Content of Wheat Seedlings Under Salt Stress

**FIGURE 8:**
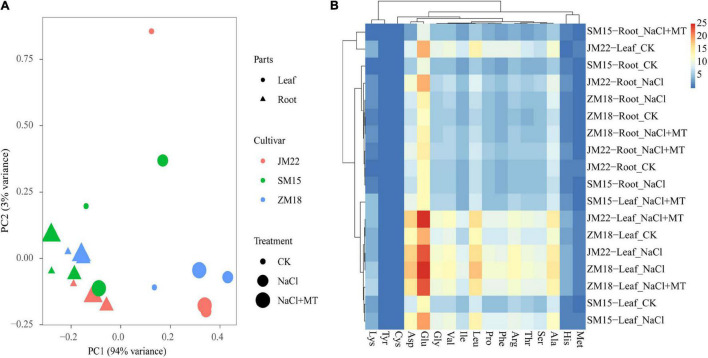
The principal component analysis (PCA) **(A)** and cluster heatmap **(B)** of amino acid content of wheat seedlings under different treatments. Asp, aspartic acid; Glu, glutamic; His, histidine; Ser, serine; Arg, arginine; Gly, glycine; Thr, threonine; Pro, proline; Ala, alanine; Val, valine; Met, methionine; Cys, cysteine; Ile, isoleucine; Leu, leucine; Phe, phenylalanine; Lys, lysine; Tyr, tyrosine.

Figure 8 shows the variations in amino acid content in plants of three wheat varieties under different treatments. Principal component analysis extracted two major components that together accounted for 97.0% of the variance in the dataset. Principal component 1 (PC1, *x*-axis) explained 94.0% of the variation among the individual samples, and principal component 2 (PC2, *y*-axis) explained 3.0% of the variation. Under NaCl stress, the amino acid content in the roots and leaves of three cultivars shifted greatly along PC1 axis (Figure 8A). Glu content showed high content, but Met, Cys, His, and Tyr content were low in plants. Cys and Met content in the root of JM 22 and Cys content in the root of SM15 and ZM18 decreased under salt stress, but increased when MT application. Lys and Tyr content in leaf both increased under salt stress and further increased with MT application; His content in leaf increased significantly under both NaCl and NaCl-MT treatments. Other amino acids increased under NaCl and decreased under NaCl-MT without recovering to the level of control (Figure 8B and Supplementary Table 1).

## Discussion

**FIGURE 9:**
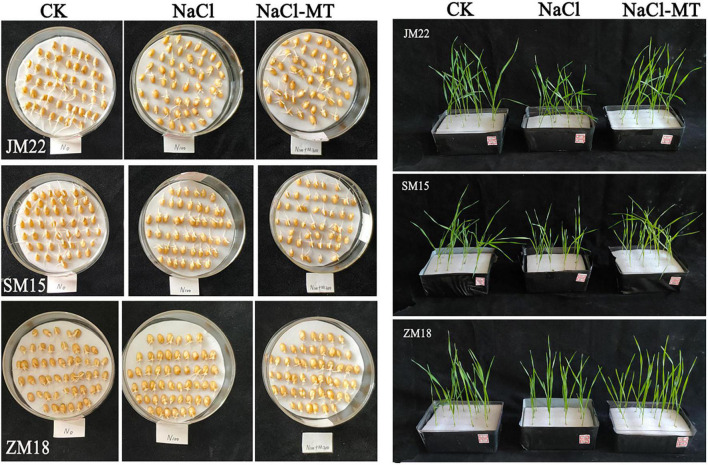
Schematic diagram of seed germination and seedling growth with MT under salt stress.

Salinity, one of the major abiotic stresses, limits the growth and productivity of many field crops. For increasingly extreme climate events, it is becoming the most important scientific research and agricultural practices in improving the salt tolerance of crops and exploiting arable areas of saline soils (Zhang et al., 2011; Himabindu et al., 2016). MT plays significant roles in antisenescence and antistress (Arnao and Hernández-Ruiz, 2014). It is well documented that exogenous MT can also improve the salinity resistance of wheat seedlings (Ke et al., 2018; Liu et al., 2020). During seed germination, exogenous MT (20 and 1 μM) pretreatment enhanced the germination rate of cotton, wheat, cucumber, and so on (Zhang N. et al., 2014; Ke et al., 2018; Chen et al., 2021). In our result, however, the germination rate showed somewhat improvement on ZM18 and SM15, no improvement on JM22, and furtherly inhibited the length of radical and germ under salt stress (Table 2 and Figure 9). On the one hand, different MT concentrations for different crops might be partly reasons. However, concentration was not the main and only reason because MT (0–500 μM) pretreatment recovered root vigor and growth of two maize varieties and decreased relative electrolytic leakage in roots and leaves during the period of seed germination and seedling cultivation under NaCl stress (Li X. et al., 2017). This is likely for different treatment modes, plant pretreated with MT often shows better stress resistance even under lower concentrations, but MT was added directly to the culture solution especially in high concentrations, the seed germination was inhibited, and the growth of radical and radicle appeared retardation because plants would trigger a defensive response as external stress elements. On the other hand, the radicle number increased significantly under salt and MT condition, which suggested that MT promoted lateral root produced and growth of plants under NaCl stress. This result was consistent with the newest report that MT promotes lateral root under salt stress (Javeed et al., 2021).

Exogenous MT partially mitigated the salt stress-induced inhibition of whole-plant growth by reducing the accumulation of H_2_O_2_ in wheat leaf and increasing endogenous MT content and polyamine contents (Ke et al., 2018). Rice and cotton plants pretreated with MT also reduced H_2_O_2_ contents in leaf and roots, which accumulate high H_2_O_2_ concentration induced by salt stress (Li X. et al., 2017; Jiang et al., 2021). In our research, the changing rule of H_2_O_2_ content in root was also decreased with salt and MT treatments compared with NaCl alone treatment. However, H_2_O_2_ content in wheat leaf was significantly decreased under salt stress alone compare with CK, and MT application did not significantly recover H_2_O_2_ content to the level of control (Figure 7). The possible reason was that roots receive stress signals to turn on the defense system including recognizing and appointing exogenous MT as a stress regulator in a short time; for leaf, there was delayed effects for that MT has been reported as a potent long-distance signal to translocate stress message from roots to leaf (Li H. et al., 2017). Whether seeds of rice and wheat were pretreated with MT for 3–7 days, or the cotton seedling was treated with foliar spray MT every 24 h for 12 days, plants have been finished antistress training (Li X. et al., 2017; Ke et al., 2018; Jiang et al., 2021). Besides playing a vital signaling role during stress responses, H_2_O_2_ was induced by respiratory burst oxidase homologs (RBOH) to convert eventually into other ROS (OH) by the Fenton reaction and Haber–Weiss reaction (Liu et al., 2020; Michard and Simon, 2020). Additionally, MT treatment enhanced significantly the activities of antioxidant enzymes (especially SOD and CAT) and decreased MDA content in leaves resulted in a decrease in H_2_O_2_ level, which was conformed to this conclusion of Li X. et al. (2017). Not only does MT act as an endogenous antioxidant that enhanced the antioxidant capacity, but also MT was applied exogenously to modulate subcellular antioxidant systems in barley (Li et al., 2016). The possible response mechanism is still unknown comprehensively considering systematic reaction and crosstalk between organs.

Another effective strategy to resist salt stress employed by plants is to keep ion homeostasis and relieve ionic toxicity. One important approach that increases salt tolerance is to maintain a high level of [K^+^]/[Na^+^] ratio in cells (Flowers and Läuchli, 1983). Many of glycophytes are subjected to NaCl stress which showed that Na^+^ in roots and leaves significantly increased, and K^+^ content clearly decreased compared with those of the control (Li X. et al., 2017; Ren et al., 2020). Na^+^ is sensed possibly by Na^+^ sensor glucuronosyltransferase, activates Ca^2+^ channels, and increases Ca^2+^ influx into the cytosol (Jiang et al., 2019). In our study, MT application significantly decreased Na^+^ contents, increased [K^+^]/[Na^+^] ratio in root induced by NaCl stress alone, but further increased Na^+^ content, and decreased [K^+^]/[Na^+^] in stem and leaf. This suggested that MT may take ameliorating effect first on the root, and meantime, a higher Na ion concentration formed along with nutrient solution flows and circulates in stem and leaf. It is known that the ion transport system including ionic equilibrium of [K^+^] and [Na^+^] and [K^+^]/[Na^+^] is often considered in a signaling network involving H_2_O_2_ and Ca^2+^. Kaya et al. (2019) reported that MT treatments increase plant growth attributes to increased Ca^2+^ and K^+^ in the leaves and reduced MDA, H_2_O_2_ in Cd-stressed wheat plants. MT also helps cold-stress plants that possessed higher Ca^2+^-ATPase, which are important for the ATP formation (Sun et al., 2018). In our results, SM15 also showed a higher Ca^2+^ keeping ability in stem and leaf under salt stress alone; ZM 18 was more easily adjusted by MT to increase significantly Ca^2+^ content in root and leaf under salt stress. These results conform to that the increase in Ca^2+^ content in cytosol that triggers RBOH activity directly induces the formation of H_2_O_2_ that is eventually converted into other ROS (Michard and Simon, 2020), which is also interpreted as why H_2_O_2_ content decreased in leaves under salt treatment (Figure 7).

Salt stress promoted storage protein degradative, and thus, amino acid content changed accordingly (Zhang et al., 2017). Lysine (Lys) content is decreased with MT and transforms into other substances to raise the level of stress tolerance in wheat seeds during germination under drought stress (Li et al., 2020). MT also accelerates the metabolic flow from the precursor amino acids arginine and methionine to polyamines and decreases the degradation of salt-induced polyamines (Ke et al., 2018). From our study, the consistent results showed that salt stress increased Arg and Met contents, which decreased with MT application to some degrees. Additionally, Cys and Met contents in root of JM 22 and Cys content in the root of SM15 and ZM18 decreased under salt stress, but increased when MT application. This suggested that Cys is likely to participate in the H_2_S-Cys cycle, which enhances its roles in the regulation of the antioxidant system (Huang et al., 2021). In our study, Lys and Tyr contents in leaf both increased under salt stress and further increased with MT application; His content in leaf increased significantly under both NaCl and NaCl-MT treatments. Other amino acids increased under NaCl and decreased under NaCl-MT without recovering to the level of control. It is known that MT often helps to induce amino acid accumulation in root and leaf to enhance the cellular osmotic potential (Cui et al., 2018). Lysine has been reported to be transformed into proline, aminobutyric acid, and polyamines during drought resisting processes (Hare and Cress, 1997; Klessig et al., 2000). So, our results also provided the evidence that MT increases the ability for the leaves of different wheat varieties to keep high water status and accumulate high soluble protein, soluble sugar, and proline content under NaCl stress (Table 3 and Figures 4, 5). On the other hand, lysine residues on histone terminus are deacetylated by histone deacetylase 14, which is involved in the biosynthesis of MT in *Arabidopsis thaliana* (Zhao et al., 2018). Both Lys and His contents increased in leaf under NaCl and NaCl + MT which indicated a complex biochemical process, during which the MT content changing the interaction between MT and amino acid, and involved mechanism would be the next important research point.

It is noted that MT application methods (pretreated coating or soaking, leaf pray, root or rhizospheric application, mixed application with other growth regulators) and varied experimental elements (MT concentration, plant species, varieties, and adversity types) appear to have different regulatory effects during stress-resistance process (Kostopoulou et al., 2015; Jiang C. et al., 2016; Li X. et al., 2017). In our research, different wheat varieties showed different salt-sensitivities. JM22, a widely adaptive super-high-yield variety, was not salt-resistant and shows an obvious decrease on germination rate and activity of SOD and CAT under NaCl stress. SM15 and ZM18 showed higher salt-resistant, considering that SOD activity of SM15 did not decrease but increased significantly and higher germination rate and proline accumulation under NaCl stress alone. MT at a concentration of 300 μmol L^–1^ played roles as regulated antioxygen to keep the physiological equilibrium of wheat seedling and to promote germination and lateral roots, but it took an inhibitory effect on the length of radicle and germ. The optimal concentration of MT on wheat seed germination and growth of wheat seedling and the dominant varieties of wheat for coping with salt stress should be further researched for meeting future production reality needs.

## Conclusion

Exogenous MT promoted germination rate of SM15 and ZM 18 recovering control level under salt stress, but had no effect on that of JM22. SM15 and ZM 18 showed higher salt resistance than that of JM22, especially for SM15 that had higher proline content, root vigor, and higher antioxygen system under salt stress. JM22 was salt-sensitive. MT at 300 ’μmol L^–1^ inhibited the length of germ and radicle, but promoted lateral root production of seed embryo, and increased shoot and root length of wheat seedling under NaCl stress. Salt stress-induced antioxygen system activity enhancing and H_2_O_2_ content increasing, decreased soluble protein, soluble sugar content, [K^+^]/[Na^+^] in leaf and root vigor, and increased Na^+^ content in root and leaf. MT application could increase proline content, soluble protein, soluble sugar, Ca^2+^ content, and vital amino acid content in leaf to keep high water status and maintain a low level of H_2_O_2_ content and [K^+^]/[Na^+^] ratio in leaf. MT increased root vigor, [K^+^]/[Na^+^] ratio, and decreased H_2_O_2_ content in root induced by salt stress. The findings of this study showed that the root as the first defense organization responded to salt stress was rapidly and earlier than the leaf in a short time (24 h), and the signal transmission and interaction between roots and leaf maintained to reduce stress injury as much as possible. So, in wheat production, the optimal concentration, varieties, and MT application method should be considered comprehensively at the sowing and seedling stages.

## Data Availability Statement

The raw data supporting the conclusions of this article will be made available by the authors, without undue reservation.

## Author Contributions

DL and RL initiated and designed the research. ZZ, HL, XF, and SZ performed the experiments and collected the data. XZ, NY, and JS wrote the code and tested the methods. LL, ZZ, and DL analyzed the data and wrote the manuscript. All authors contributed to the article and approved the submitted version.

## Conflict of Interest

The authors declare that the research was conducted in the absence of any commercial or financial relationships that could be construed as a potential conflict of interest.

## Publisher’s Note

All claims expressed in this article are solely those of the authors and do not necessarily represent those of their affiliated organizations, or those of the publisher, the editors and the reviewers. Any product that may be evaluated in this article, or claim that may be made by its manufacturer, is not guaranteed or endorsed by the publisher.
